# The consequences of data dispersion in genomics: a comparative analysis of data sources for precision medicine

**DOI:** 10.1186/s12911-023-02342-w

**Published:** 2023-11-09

**Authors:** Mireia Costa, Alberto García S., Oscar Pastor

**Affiliations:** PROS Research Center, VRAIN Research Institute, Universitat Politècnica de València, Camino de Vera, Valencia, Spain

**Keywords:** Precision medicine, DNA variations, Concordance, Completeness, Genomic data sources

## Abstract

**Background:**

Genomics-based clinical diagnosis has emerged as a novel medical approach to improve diagnosis and treatment. However, advances in sequencing techniques have increased the generation of genomics data dramatically. This has led to several data management problems, one of which is data dispersion (i.e., genomics data is scattered across hundreds of data repositories). In this context, geneticists try to remediate the above-mentioned problem by limiting the scope of their work to a single data source they know and trust. This work has studied the consequences of focusing on a single data source rather than considering the many different existing genomics data sources.

**Methods:**

The analysis is based on the data associated with two groups of disorders (i.e., oncology and cardiology) accessible from six well-known genomic data sources (i.e., ClinVar, Ensembl, GWAS Catalog, LOVD, CIViC, and CardioDB). Two dimensions have been considered in this analysis, namely, completeness and concordance. Completeness has been evaluated at two levels. First, by analyzing the information provided by each data source with regard to a conceptual schema data model (i.e., the schema level). Second, by analyzing the DNA variations provided by each data source as related to any of the disorders selected (i.e., the data level). Concordance has been evaluated by comparing the consensus among the data sources regarding the clinical relevance of each variation and disorder.

**Results:**

The data sources with the highest completeness at the schema level are ClinVar, Ensembl, and CIViC. ClinVar has the highest completeness at the data level data source for the oncology and cardiology disorders. However, there are clinically relevant variations that are exclusive to other data sources, and they must be considered in order to provide the best clinical diagnosis. Although the information available in the data sources is predominantly concordant, discordance among the analyzed data exist. This can lead to inaccurate diagnoses.

**Conclusion:**

Precision medicine analyses using a single genomics data source leads to incomplete results. Also, there are concordance problems that threaten the correctness of the genomics-based diagnosis results.

## Background

### Introduction

Precision medicine has emerged as a novel medical approach, transforming traditional reactive medicine into a more proactive, patient-centered approach. One of the several artifacts for delivering individualized treatment is genomics-based clinical diagnosis, which sequences and analyzes the genome to discover patients’ predispositions to disorders, altered drug responses, or the origin of a disease [1, 2].

The advent of Next Generation Sequencing (NGS) techniques has significantly reduced the time and cost of genome sequencing [3]. However, genomics has become a big data problem, and developing an effective data management strategy remains a challenge. This situation is exacerbated by four factors; the first factor is the vast amount of genomics data publicly available, as well as the rapid rate at which we generate it. The pace at which new genomics knowledge emerges is the second factor; because of the ever-increasing amount of publicly available genomics data, genomics knowledge constantly evolves and changes, resulting in high variability over time [4]. The third factor is the significant dispersion of genomics knowledge; the available knowledge is dispersed across hundreds of data sources that diverge in content, size, format, and structure [5]. The fourth factor is the necessity for interoperability among genomics data sources. As previously stated, genomics information is widely dispersed, which causes it to be either isolated (i.e., only available in one database) or represented in a wide variety of notations and formats in the databases, resulting in integration issues.

In summary, the current genomics big data problem is caused by a massive amount of available data, the constant evolution of genomics knowledge, knowledge dispersion across hundreds of data sources, and a lack of interoperability. These issues must be addressed if accurate genomics-based clinical diagnoses are to be achieved. Integrating and analyzing existing knowledge across genomics data sources is critical in guaranteeing that all available knowledge is captured. However, this is not common practice, and genomics experts frequently limit the scope of their work to a single data source they know and trust, causing them to miss relevant knowledge and fail to detect potentially relevant data concordance inconsistencies.

The purpose of this research is to evaluate the consequences of failing to consider multiple genomics data sources in genomics-based clinical diagnoses. We conducted a comparative analysis of information from various well-known data sources about DNA variations associated with oncology and cardiology disorders. We used a data quality perspective to analyze a group of well-known genomics data sources for oncology and cardiology disorders. In particular, we evaluated two metrics (concordance and completeness).

### Related work

There is very little literature describing comparative analyses of genomics data sources. Currently, no studies are describing a comparative analysis of the available information for DNA variations associated with cardiology disorders. Still, there are some studies regarding variations related to oncology disorders. These studies have two major limitations. First, they only compare technical aspects and the type of information provided rather than the data provided by each data source. Second, they only compare data sources that only host oncology-related data. These studies are highlighted below.

Borchert et al. analyzed genomics data sources containing information on DNA variations associated with oncology disorders. Several technical characteristics were identified in the study, including license, accessibility, update frequency, percentage of somatic versus germline variations, and evidence tiers [6]. The authors compared each data source based on these characteristics but did not perform a direct comparison of the information provided by each source.

Yu et al. took a similar approach to Borchert et al. in their research, comparing a new data source called PreMedKB to other existing data sources that provide oncology-related information [7]. Unlike Borchert et al.’s work, which focused solely on technical characteristics such as license, accessibility, and update frequency, Yu et al. also considered term normalization, search methods, data structure, and visual representation. Notably, Yu et al. also ignored the actual data provided by each source in their analysis. Instead, they focused on the technical characteristics that affect the usability and reliability of the information.

Li et al. examined and classified twenty-four data sources containing oncology-related information [8]. Like the two previous studies, their work aimed to provide a high-level description of the data sources rather than a comparative analysis of the information they contained. Their research thoroughly understood the types of data stored in each source, including data format, source quality, and target users.

The only study that closely resembles the approach taken in this work is that of Pallarz et al. [9], where the role of DNA variations in oncology disorders was compared using data from seven oncology-specific databases: CIViC, OncoKB, Cancer Gene Census, Database of Curated Mutations, and CGI Biomarkers. Their findings revealed that while the information provided by each data source had significant overlap, each source also contained unique information. Furthermore, no single data source supplanted the others, emphasizing the importance of combining data from various sources to understand the role of DNA variations in oncology disorders. The findings of this study highlighted the importance of continuing research into the comparative analysis of oncology-related data sources.

This comparative genomics data analysis adds to the small body of literature on the subject. As demonstrated by the studies mentioned above, most publications in this area focus either on disease-specific data sources or technical aspects rather than analyzing the data contained within these sources. This work is unique in two ways. First, we considered data sources for both disease-specific and general-purpose genomics (see Data sources). Second, we thoroughly examined the data contained in these sources, providing valuable insights into the potential clinical significance of DNA variations in oncology and cardiology disorders. This enabled us to provide a holistic view of the available data in the fields of cardiology and oncology.

## Methods

**Fig. 1:**
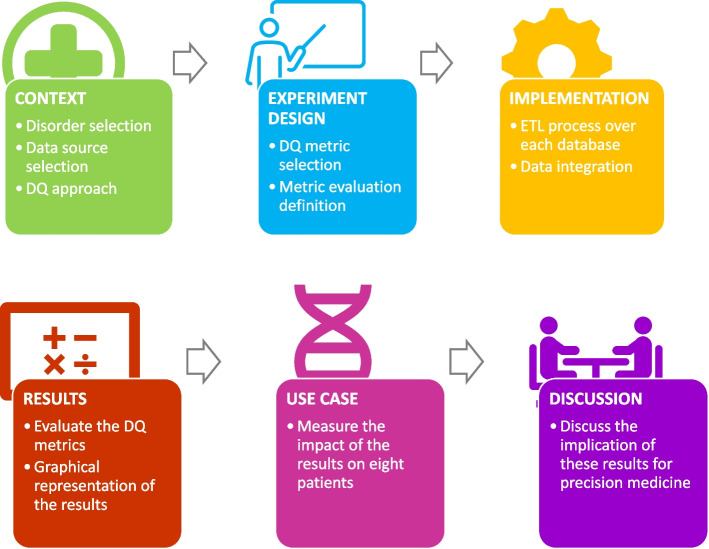
Pipeline followed for performing the comparative analysis of genomic data sourcess

To conduct the comparative study, we followed the pipeline depicted in Fig. 1. This comparative analysis focused on studying the consequences of data dispersion. To do so, we examined data from five cardiology and five oncology disorders using a variety of well-known genomics data sources. Then, the experiment was designed by selecting the best data quality (DQ) metrics for measuring data dispersion and developing a procedure for evaluating them. To support this evaluation, we implemented an ETL procedure to gather the DNA variations associated with the selected disorders from each data source, a data integration procedure to integrate all the variations related to each disorder, and a set of scripts for the metric evaluation. These technological implementations facilitated the evaluate the DQ metric and the visual representation of the results. Finally, we evaluated the effects of data dispersion via a use case with eight real patients.

This Section describes the context, the experiment design, and the implementation phases while Results section shows the results, Use case focuses on the use case, and Conclusions discusses our findings.

### Context

#### Disorders

This analysis studied DNA variations assocaited with five oncology disorders (see Table 1) as well as five cardiopathy disorders (see Table 2). The disorder’s name, its ID in the Human Phenotype Ontology (HPO), and a brief description is shown on each table.

**Table 1:** The oncology disorders selected for the comparative analysis

Name	HPO ID	Description
Acute Lymphoblastic Leukemia	HP:0006721	Malignant hematopoietic disorder affecting the bone marrow and the peripheral blood, and characterized by excess lymphoblasts.
Acute Myeloid Leukemia	OMIM:601626	A group of neoplasms arising from precursor cells committed to the myeloid cell-line differentiation.
Neuroblastoma	HP:0003006	A solid tumor that originates in neural crest cells of the sympathetic nervous system.
Retinoblastoma	OMIM:180200	A malignant tumor that originates in the nuclear layer of the retina.
Osteosarcoma	HP:0002669	A malignant bone tumor that tends to develop during adolescence and usually affects the long bones.

**Table 2:** The cardiology disorders selected for the comparative analysis

Name	HPO ID	Description
Arrhythmogenic Cardiomyopathy	HP:0011663	A progressive replacement of right ventricular myocardium with adipose and fibrous tissue.
Dilated Cardiomyopathy	HP:0001644	A left ventricular dilatation and left ventricular systolic dysfunction
Hypertrophic Cardiomyopathy	HP:0001639	An increased ventricular wall thickness or mass.
Restrictive Cardiomyopathy	HP:0001723	A ventricular filling pattern with increased myocardium stiffness.
Short QT Syndrome	ORPHA:51083	A short QT interval on an EKG that does not significantly change with heart rate, tall and peaked T waves, and a structurally normal heart.

#### Data sources

The data compared in this study was collected from six of the most well-known and widely used genomics data sources. Four of them are general-purpose, meaning they contain information on various disorders. The other two data sources are disease-specific, meaning they only store information about specific disorders. Given the disorders under consideration in this study, one data source is dedicated to oncology and the other to cardiology.

The general-purpose data sources are ClinVar, GWAS Catalog, Ensembl, and LOVD. ClinVar [10] is a public archive that contains information about the role of genetic variations in clinical disorders. GWAS Catalog [11] provides data about GWAS studies that seek to determine whether a genetic variation is statistically associated with the risk of developing a particular disorder. Ensembl [12] aggregates information from various data sources, including ClinVar and GWAS Catalog. LOVD [13] is composed of several gene-specific data sources containing information about variation-disorder clinical relationships.

For the disease-specific data sources, we considered CIViC [14] and CardioDB [15]. CIViC provides information about the role of variations in oncology disorders at various clinical dimensions. CardioDB is an expert-curated data source that contains information about the variations associated with three specific cardiology disorders, namely, Arrhythmogenic Cardiomyopathy, Hypertrophic Cardiomyopathy, and Dilated Cardiomyopathy.

#### DQ approach

Experts in genomics analyze genomic data sources on a regular basis to choose the most appropriate diagnoses and therapies for their patients. Assuring the quality of the data in these sources is crucial in this situation to prevent risk to any patient’s health [16].

Despite the fact that many authors emphasize the importance of data quality in genomics [17, 18] and healthcare in general [16], there is no standardized paradigm for evaluating data quality in these scenarios.

However, a recently-published comprehensive literature review on this topic proposes seven DQ metrics as a standard for healthcare [19]: Completeness [20], Correctness [20], Currency [21], Concordance [22], Usability [19, 21], Relevance [20], and Duplication [19]. More details regarding these metrics in Table 3.

**Table 3:** DQ metrics for Helthcare

Name	Description
Completeness	The extent to which data are in sufficient breadth, depth, and scope for the task at hand.
Correctness	The extent to which the data are correct, reliable, and free of error.
Currency	The extent to which data is sufficiently up-to-date for the task at hand.
Concordance	The degree of agreement or compatibility between data elements.
Usability	The extent to which the data is understandable and accessible.
Relevance	The extent to which the data are applicable and useful for the task at hand.
Duplication	Existence of multiple existences of the same data entity.

### Experiment design

This section covers all of the relevant aspects concerning the experiment design.

#### DQ metric selection

As previously stated, the purpose of this study is to quantify the consequences of failing to use multiple genomics data sources when making clinical diagnoses based on genomics data. In this situation, two significant issues arise. The first issue is that not all relevant data about a disorder is available in a single data source. The second issue is that concordance inconsistencies in the data may not be identified.

As a result of the two issues mentioned above, we focused our efforts on two DQ metrics: completeness and concordance. Through the completeness study, we sought to determine whether relying on a single data source was sufficient for capturing all relevant information. The concordance analysis allowed us to assess current knowledge’s consistency and decide whether it was acceptable to make clinical decisions using only one genomics data source.

#### Metric evaluation definition

This section describes how each metric was evaluated. The metric of completeness was evaluated at two levels: schema and data [21]. Completeness at the Schema Level (CSL) measures how well the concepts required to represent variation information are represented in the data source schema. As a result, CSL evaluation is unaffected by the disorder under investigation. We used the Conceptual Schema of the Human Genome -CSHG- (more details in the following section) to calculate the CSL, which helped us identify the entities a data source should represent in its schema.

The CSL of each data source was calculated as the ratio of the number of CSHG entities represented to the total number of entities in the CSHG (see Eq. 1).

1 $$\begin{aligned} CSL= \frac{\textit{Number of entities of the CSHG represented}}{\textit{Total number of entities in the CSHG}} \end{aligned}$$

The Completeness at the Data Level (CDL) metric assesses whether a particular data source contains every variation associated with a specific disorder reported in other data sources. Unlike the CSL metric, this metric relies on both the data source and the disorder under investigation. CDL was calculated by dividing the number of variations in a data source by the total number of unique variations in all data sources (see Eq. 2).

2 $$\begin{aligned} CDL= \frac{\textit{Number of variations in the data source}}{\textit{Number of unique variations in all the data sources}} \end{aligned}$$

The concordance metric determines whether all data sources show consistent associations between a variation and a specific disorder. Otherwise, the information is considered discordant (i.e., contradictory). This variation-disorder association is represented by either an “interpretation” approach, in which geneticists determine whether the variation causes or does not cause the disorder, or a “statistical association” approach, in which the strength of the association between the variation and the disorder is calculated.

For the “interpretation” approach, the association between a variation and a disorder is considered concordant if every interpretation available for such a variation reports the same disorder-causing effect. For instance, if two interpretations agree that a variation causes a disorder (i.e., the variation is pathogenic or likely pathogenic). In contrast, the information is discordant if one interpretation considers the variation to be disorder-causing and the other does not (i.e., the variation is interpreted as benign or likely benign).

For the “statistical metrics” approach, we followed the recommendations made by our clinical partners. The association between variation and disorder is considered relevant if the p-value is less than $5x10^{-8}$ and the confidence interval (CI) does not cross one. Thus, when all available GWAS studies agree on whether or not the variation-disorder association is relevant, the variation is considered concordant.

3 $$\begin{aligned} Concordance= \frac{\textit{Number of variations with concordant information}}{\textit{Total number of unique variations in all the data sources}} \end{aligned}$$

#### Data model

**Fig. 2:**
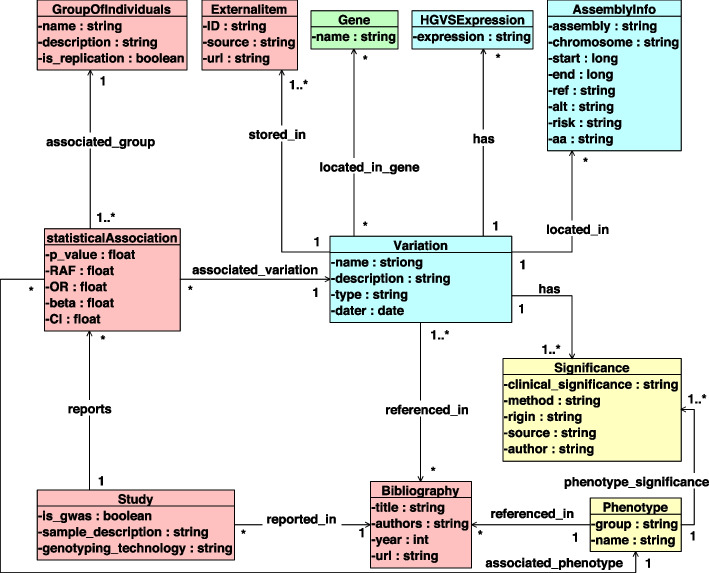
Subschema of the CSHG obtained after carrying out the ISGE method. The variation view is depicted in blue. The evidence view is depicted in red. The phenotype view is depicted in yellow. The structural view is depicted in green

The CSHG [23, 24] was designed to represent genomic information in its various dimensions. The ISGE method [25] was used to instantiate a subschema of the CSHG containing only the relevant information for the study of DNA variations. This new subschema (shown in Fig. 2) is divided into four views that structure all relevant details on DNA variations:

- The *Structural view* contains the structural components of the human genome. It consists of the Gene entity, which represents the information about the genes where DNA variations are located.
- The *Variation view* describes changes that occur in our genome (i.e., DNA variations). It consists of the Variation, HGVSExpression, and AssemblyInfo entities. The Variation entity represents the intrinsic information about a variation. The HGVSExpression is the formal description of the HGVS notation, which is a standard used to represent changes at the DNA, RNA, or amino acid level. It describes the change and its location. Several HGVSExpression can be associated with a Variation. Finally, the AssemblyInfo entity provides information about a Variation in the human genome (i.e., its position and alleles for a given coordinate system, called assembly).
- The *Evidence view* models the statistical metrics information. It represents the information used for establishing variation-disorder associations, including genomics studies, relevant bibliography, statistical associations, and external sources. It consists of the GroupOfIndividuals, ExternalItem, StatisticalAssociation, Study, and Bibliography entities. The Bibliography entity represents existing literature with information about a variation. The Study entity represents different studies performed on a group of people for a given DNA variation (e.g., a GWAS study). The StatisticalAssociation entity represents the statistical metrics that result from a study. Finally, the GroupOfIndividuals entity represents the characteristics of the group of individuals for which the statistical association has been calculated.
- The *Phenotype view* models the interpretation approach. It details the two most relevant concepts about variations and their association with disorders: the Phenotype and the Significance entities. The Phenotype entity represents the disorder associated with the variation. The Significance entity represents the interpretation or role of variation for a specific disorder.

This subschema served as a framework for determining whether a data source contains all the entities required to study variations correctly and measure the CSL metric.

### Experiment implementation

An ETL process, a data integration process, and a DQ metrics evaluation process are the three technological implementations that aid in evaluating the DQ metrics. These three processes were developed using the R programming language.

We obtained all variations associated with the five oncology and five cardiology disorders we selected using the ETL process. During the extraction stage, we implemented a connection mechanism to each data source and the necessary filtering strategies in order to acquire only those variations related to the phenotype under investigation. During the transformation stage, we transformed the data to follow a JSON format with a structure that adheres to the data model described in Data model.

A JSON file was generated for each data source and disorder. These files were consolidated into a single file in order to determine whether a variation was present across multiple data sources. This produced a JSON file containing a single instance of each variation associated with a given disorder. Finally, algorithms were developed to automate the evaluation of DQ metrics (see formula in Metric evaluation definition).

## Results

This section presents the results of evaluating the completeness and concordance metrics. First, Completeness at the schema level shows the CSL evaluation results for each of the six data sources examined in our study. Then, Completeness at the Data Level illustrates the CDL analysis results for the oncology and cardiology disorders. Finally, Concordance presents the results of the concordance analysis for both groups of disorders.

### Completeness at the schema level

We examined the entities of the CSHG subschema that each data source represents in its internal schema to obtain the CSL results. ClinVar, Ensembl, and CIViC contain information on ExternalItem, Gene, HGVSExpression, Variation, AssemblyInfo, Significance, Phenotype, and Bibliography. LOVD and CardioDB, except for the HGVSExpression entity, contain information on the same entities mentioned above. These five data sources have one thing in common: they don’t store information about GWAS studies. As a result, the GroupOfIndividuals, StatisticalAssociation, and Study entities are not considered in their internal schema.

In the case of the GWAS Catalog, the CSHG entities represented in its database schema are those that allow characterizing the information about the GWAS studies: the GroupOfIndividuals, StatisticalAssociation, Study, Bibliography, Phenotype, Gene, and Variation entities.

Considering all the above, we applied Eq. 1 to calculate the CSL of each data source. Table 4 summarizes the findings.

**Table 4:** The results for the completeness at schema level for ClinVar, Ensembl, LOVD, CIViC, CardioDB and GWAS Catalog. The results have been calculated according to Eq. 1

Database	Number of entities	Completeness at the Schema Level (%)
ClinVar	8	72.72%
Ensembl	8	72.72%
LOVD	7	63.63%
CIViC	8	72.72%
CardioDB	7	63.63%
GWAS Catalog	7	63.63%

### Completeness at the Data Level

In order to calculate the results for the CDL, we first studied the variations associated with oncology disorders; we compared the variations provided by each general-purpose data source and the cancer-specific data source for each of the five oncology disorders. Then, we compared the variations associated with cardiology disorders provided by each general-purpose data source and the cardiology-specific data source for the five cardiology disorders. Completeness at the Data Level in oncology disorders and Completeness at the Data Level in cardiology disorders report the results of the CDL for the oncology and cardiology disorders, respectively.

#### Completeness at the data Level in oncology disorders

As described in Methods, we studied the variations provided by the general-purpose data sources (i.e., ClinVar, Ensembl, GWAS Catalog, and LOVD) and the oncology-specific data source (i.e., CIViC) associated with five oncology disorders, namely, Acute Myeloid Leukemia, Neuroblastoma, Osteosarcoma, Retinoblastoma, and Acute Lymphoblastic Leukemia.

**Fig. 3:**
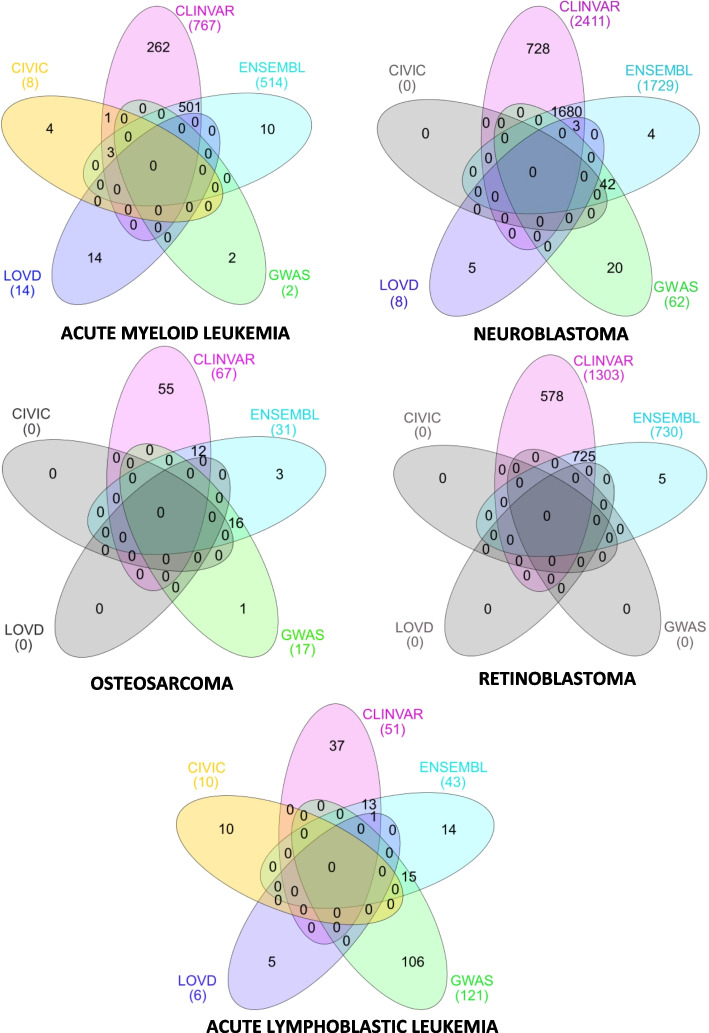
Number of variations per database and oncology disorder. In yellow, the variations from CIViC. In blue, the variations from Ensembl. In purple, the variations from LOVD. In pink, the variations from ClinVar. In green, the variations from GWAS Catalog. In white, the data sources without variations

Figure 3 depicts the distribution of variations by data source and disorder using Venn diagrams. The distribution of variations is significantly heterogeneous among the five disorders. The only disorders for which all data sources report associated variations are Acute Myeloid Leukemia and Acute Lymphoblastic Leukemia. On the contrary, Retinoblastoma has the fewest data sources with associated information, with only two of them providing associated variations.

ClinVar and Ensembl are the only data sources that report variations associated with all the studied disorders. For the other data sources, the GWAS Catalog contains information about four disorders, LOVD about three, and CIViC about two.

Each data source has unique variations not found in the others (i.e., *exclusive variations*). For all disorders except for Acute Lymphoblastic Leukemia, the data source that provides the most exclusive variations is ClinVar. The GWAS Catalog data source provides the most exclusive variations for Acute Lymphoblastic Leukemia.

The most significant data overlap is between ClinVar and Ensembl, followed by the one between the GWAS Catalog and Ensembl. This was expected because Ensembl incorporates information from these two data sources. Other minor overlaps exist. There is an overlap in Acute Myeloid Leukemia information between ClinVar, CIViC, and Ensembl or in Neuroblastoma and Acute Lymphoblastic Leukemia data between LOVD, ClinVar, and Ensembl.

Table 5 summarizes the CDL results obtained using the formula in Eq. 2. Because of the numerous exclusive variations it reports, ClinVar has the highest CDL of any data source. On the contrary, LOVD and CIViC have the lowest CDL for almost all disorders. Nevertheless, because none of the data sources includes every variation, those with low CDL should be kept because they have unique variations that can be clinically significant.

**Table 5:** The results for the completeness at data level for oncology disorders. Equation 2 was used to calculate these results. Note that the total number of unique variations does not equal the total number of variations stored in each database. This is because the same variation can be found in multiple databases (see Fig. 3)

Database	Number of variations	Completeness at the Data Level (%)
Acute Lymphoblastic Leukemia		
ClinVar	51	25.37%
Ensembl	43	% 21.39
GWAS Catalog	144	71.64%
LOVD	6	2.98%
CIViC	10	4.98%
Number of unique variations	201	
Acute Myeloid Leukemia		
ClinVar	767	96.23%
Ensembl	514	64.49%
GWAS Catalog	2	0.25%
LOVD	14	1.76%
CIViC	8	1.00%
Number of unique variations	797	
Neuroblastoma		
ClinVar	2,411	97.14%
Ensembl	1,729	69.66%
GWAS Catalog	62	2.50%
LOVD	8	0.32%
CIViC	0	0.00%
Number of unique variations	2,482	
Retinoblastoma		
ClinVar	1,303	99.62%
Ensembl	730	55.81%
GWAS Catalog	0	0.00%
LOVD	0	0.00%
CIViC	0	0.00%
Number of unique variations	1,308	
Osteosarcoma		
ClinVar	67	77.01%
Ensembl	31	35.63%
GWAS Catalog	17	19.54%
LOVD	0	0.00%
CIViC	0	0.00%
Number of unique variations	87	

#### Completeness at the Data Level in cardiology disorders

As described in Methods, we studied the variations provided by general-purpose data sources (i.e., ClinVar, Ensembl, GWAS Catalog, and LOVD) and cardiology-specific data sources (i.e., CardioDB) about five cardiology disorders, namely, Arrhythmogenic Cardiomyopathy, Hypertrophic Cardiomyopathy, Restrictive Cardiomyopathy, Short QT Syndrome, and Dilated Cardiomyopathy.

**Fig. 4:**
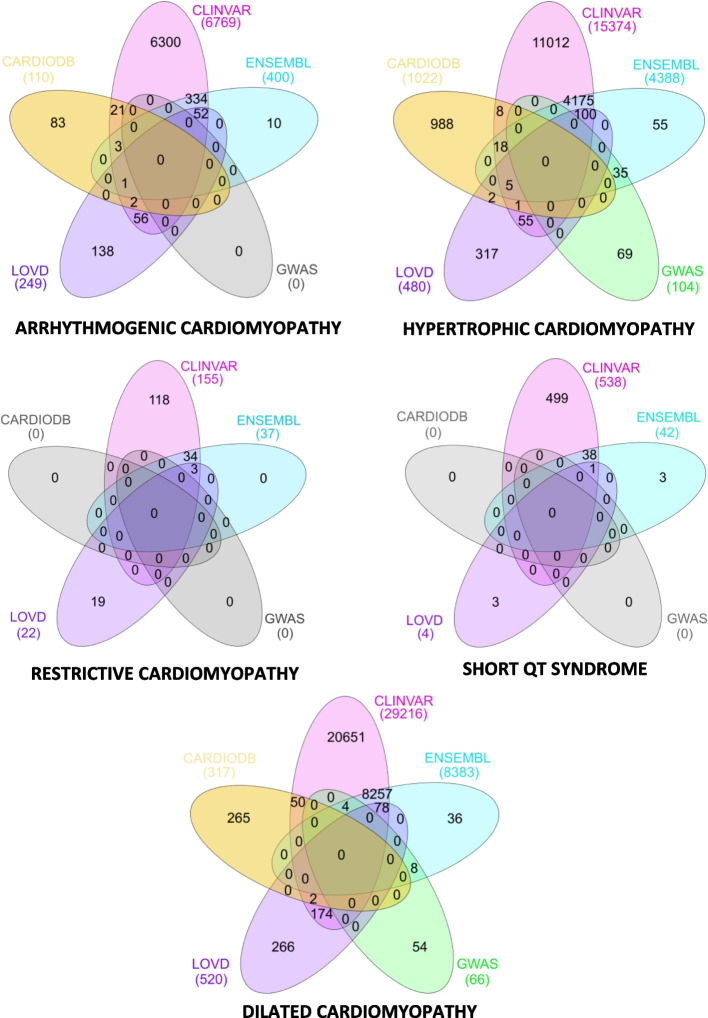
Number of variations per database and cardiology disorder. In yellow, the variations from CardioDB. In blue, the variations from Ensembl. In purple, the variations from LOVD. In pink, the variations from ClinVar. In green, the variations from GWAS Catalog. In white, the data sources without variations

The distribution of variations by data source and disorder is depicted by a Venn diagram (see Fig. 4). The distribution of variations differs significantly across disorders. While all data sources report variations associated with Dilated and Hypertrophic Cardiomyopathy, only three data sources store variations associated with Restrictive Cardiomyopathy and Short QT Syndrome, the two conditions with the fewest variations.

Like with oncology disorders, ClinVar is the data source with the most exclusive variations. However, other databases have a large number of unique variations too. For instance, CardioDB reports 83 exclusive variations for Arrhythmogenic Cardiomyopathy and 265 exclusive variations for Dilated Cardiomyopathy.

In terms of data overlap, cardiology disorders have a high rate of overlap. However, none of the variations are reported in all data sources, and only one is found in four. ClinVar and Ensembl have the most significant overlap once again. There are also considerable overlaps between CardioDB and ClinVar, as well as LOVD and ClinVar, particularly for the Arrhythmogenic Cardiomyopathy and Dilated Cardiomyopathy disorders.

Table 6 summarizes the CDL results obtained using the formula in Eq. 2. ClinVar is, by far, the most comprehensive data source for cardiology disorders. In contrast, GWAS Catalog, LOVD, and CardioDB have the lowest CDL metric results. None of the data sources achieved a CDL of 100%, indicating that those with a low CDL may still have exclusive variations that are clinically significant.

**Table 6:** The results for the completeness at data level for cardiology disorders. Equation 2 was used to calculate these results. Note that the total number of unique variations does not equal the total number of variations stored in each database. This is because the same variation can be found in multiple databases (see Fig. 4)

Database	Number of variations	Completeness at the Data Level (%)
Hypertrophic Cardiomyopathy		
ClinVar	15,374	91.29%
Ensembl	4,388	26.06%
GWAS Catalog	104	0.62%
LOVD	480	2.85%
CardioDB	1,022	6.07%
Number of unique variations	16,840	
Dilated Cardiomyopathy		
ClinVar	29,216	97.89%
Ensembl	8383	28.07%
GWAS Catalog	66	0.22%
LOVD	520	1.74%
CardioDB	317	1.06%
Number of unique variations	29,845	
Arrhythmogenic Cardiomyopathy		
ClinVar	6,769	96.70%
Ensembl	400	5.71%
GWAS Catalog	0	0.00%
LOVD	249	3.55%
CardioDB	110	1.57%
Number of unique variations	7,000	
Short QT Syndrome		
ClinVar	538	98.90%
Ensembl	42	7.72%
GWAS Catalog	0	0.00%
LOVD	4	0.74%
CardioDB	0	0.00%
Number of unique variations	544	
Restrictive Cardiomyopathy		
ClinVar	155	89.08%
Ensembl	37	21.26%
GWAS Catalog	0	0.00%
LOVD	22	12.64%
CardioDB	0	0.00%
Number of unique variations	174	

### Concordance

In order to obtain concordance results, we measured the consensus between the associations provided by the data sources. Concordance in oncology disorders and Concordance in cardiology disorders explore the levels of concordance for oncology and cardiology disorders, respectively.

#### Concordance in oncology disorders

The results for the concordance in oncology disorders are summarized in Table 7. Clinical interpretation concordance is greater than 99% for all oncology disorders. This means that if a variation appears in multiple data sources, there is a high likelihood of agreement regarding its clinical impact on a specific disorder. Despite this, there are some variations in Acute Myeloid Leukemia and Neuroblastoma disorders with contradictory interpretations.

**Table 7:** Results for the concordance dimension associated with oncology disorders

Analysis	Number of variations	Concordance (%)
Acute Lymphoblastic Leukemia		
Concordant Interpretations	216	100.00%
Discordant Interpretations	0	0.00%
Concordant GWAS	143	99.31%
Discordant GWAS	1	0.69%
Acute Myeloid Leukemia		
Concordant Interpretations	791	99.75%
Discordant Interpretations	2	0.25%
Concordant GWAS	2	100.00%
Discordant GWAS	0	0.00%
Neuroblastoma		
Concordant Interpretations	2,477	99.72%
Discordant Interpretations	7	0.28%
Concordant GWAS	64	100.00%
Discordant GWAS	0	0.00%
Retinoblastoma		
Concordant Interpretations	1,308	100.00%
Discordant Interpretations	0	0.00%
Concordant GWAS	-	-
Discordant GWAS	-	-
Osteosarcoma		
Concordant Interpretations	87	100.00%
Discordant Interpretations	0	0.00%
Concordant GWAS	17	100.00%
Discordant GWAS	0	0.00%

In the case of Acute Myeloid Leukemia, there are two variations (GATA2:c.1061 C>T and GATA2:c.1192C>T) with contradictory interpretations. The two variations have been interpreted as both Pathogenic and Likely Pathogenic in ClinVar. In this case, the conflicting interpretations arise from discrepancies in the information provided by the same data source rather than differences among data sources. This finding adds a new dimension to the concordance problem, potentially making it even more challenging to understand the role of a variation in a specific disorder.

In the Neuroblastoma case, there are seven conflicting interpretations (KIF1:c.3787 C>T, KIF1B:c.2618C>T, ALK:c.3749T>C, ALK:c.3452C>T, ALK:c.3575G>C, ALK:3383G>C, and ALK:c.3824G>A). Six of them have discordance due to contradictory information found in ClinVar. The discordance in the other case (KIF1B:c.2618C>T) is caused by the variation being interpreted as Uncertain Significance in ClinVar and Pathogenic in LOVD. As a result, a potential misinterpretation of the variation’s role in Neuroblastoma may occur if only one of these two data sources is used.

The GWAS catalog results are generally consistent, with only one variation (IKZF1:c.*1656T>C) associated with Acute Lymphoblastic Leukemia providing contradictory information. Concordance was not calculated for Retinoblastoma because no variation is associated with it in the GWAS Catalog.

#### Concordance in cardiology disorders

Table 8 summarizes the results of the concordance metric in cardiology disorders. The clinical interpretation for cardiology disorders is significantly concordant, indicating a high likelihood of agreement on the clinical impact of variations reported across different data sources. Nevertheless, the large number of conflicting interpretations cannot be ignored. For instance, more than 120 conflicting variations are associated with Hypertrophic Cardiomyopathy, Dilated Cardiomyopathy, or Arrhythmogenic Cardiomyopathy. These discrepancies in the interpretations result from differences in the information provided by different data sources as well as differences in the information reported by a single data source.

**Table 8:** Results for the concordance dimension associated with cardiology disorders

Database	Number of variations	Concordance (%)
Hypertrophic Cardiomyopathy		
Concordant Interpretations	16,551	98.64%
Discordant Interpretations	229	1.36%
Concordant GWAS	-	-
Discordant GWAS	-	-
Dilated Cardiomyopathy		
Concordant Interpretations	29,706	99.46%
Discordant Interpretations	160	0.54%
Concordant GWAS	68	100.00%
Discordant GWAS	0	0.00%
Arrhythmogenic Cardiomyopathy		
Concordant Interpretations	6,882	98.29%
Discordant Interpretations	120	1.71%
Concordant GWAS	-	-
Discordant GWAS	-	-
Short QT Syndrome		
Concordant Interpretations	546	99.82%
Discordant Interpretations	1	0.18%
Concordant GWAS	-	-
Discordant GWAS	-	-
Restrictive Cardiomyopathy		
Concordant Interpretations	176	100.00%
Discordant Interpretations	0	0.00%
Concordant GWAS	-	-
Discordant GWAS	-	-

Let us illustrate this situation. Discordant interpretations exist for 229 variations in Hypertrophic Cardiomyopathy, with the majority of these variations located in well-known cardiomyopathy-related genes such as MYBPC3 (68 variations), MYH7 (49 variations), TNNI3 (20 variations), or TNNT2 (20 variations). For instance:

- The TPM1:c.842T>C variation is classified as Pathogenic by ClinVar and LOVD but as Uncertain Significance by CardioDB.
- The variation TNNT2:c.886C>T is classified as Pathogenic by LOVD and has discordant interpretations in ClinVar (Uncertain Significance, and Likely Pathogenic).
- The variation TNNT2:c.842A>T has conflicting interpretations in LOVD (Pathogenic and Uncertain Significance).

Furthermore, 160 variations associated with Dilated Cardiomyopathy have contradictory interpretations. Almost every discordant variation is found on either the TTN gene (83 variations) or the Antisense TTN gene (TTN-AS1, 52 variations), both of which are well-known cardiomyopathy-related genes. Most of the observed discordance results from information stored in the same data source, between “Likely Benign” and “Uncertain Significance” and between “Likely Pathogenic” and “Uncertain Significance”. For instance, the TTN:c.93166C>T variation has been interpreted as Likey Pathogenic and Uncertain Significance in LOVD.

For Arrhythmogenic Cardiomyopathy, there are 120 variations with contradictory interpretations; 83 of these 120 variations are located in the DSP gene, a relevant gene when studying cardiomyopathy disorders. Some examples that highlight these discordances are the following:

- The DSP:c.7784C>T variation is reported as Pathogenic in LOVD and as Uncertain Significance in CardioDB.
- The DSG2:c.961T>A variation is reported as Pathogenic in LOVD and Uncertain Significance in ClinVar.
- The DSP:c.7916G>A variation is reported as Benign in ClinVar and Pathogenic in LOVD.

Regarding the Short QT Syndrome, there is only one variation with contradictory interpretations (KCNJ2:c.431G>A). The discordance, in this case, is due to contradictory information provided by the ClinVar data source. Finally, no variation associated with Restrictive Cardiomyopathy has contradictory interpretations.

## Use case

According to our findings, none of the data sources under consideration are complete, and numerous variations have contradictory interpretations. The DNA sequences of nine patients (see Table 9) were examined to see if any of them carried a genetic variation with inconsistent clinical interpretation. This study allowed us to determine the true impact of these discrepancies and whether they could hinder the correct diagnosis and treatment of these nine patients.

**Table 9:** Patients studied per group of disorder. Four patients suffered from an oncology disorder, while five suffered from a cardiology disorder

Group of disorder	Disorder	Patient ID
Oncology Disorder	Neuroblastoma	Onco-1, Onco-2
	Osteosarcoma	Onco-3, Onco-4
Cardiology Disorder	Arrhythmogenic Cardiomyopathy	Cardio-1, Cardio-2
	Hypertrophic Cardiomyopathy	Cardio-3, Cardio-4, Cardio-5

First, we examined the DNA sequence of four patients who had an oncology disorder (i.e., Onco-1, Onco2, Onco-3, and Onco-4). Onco-1 and Onco-2 patients have Neuroblastoma and share an identical variation: Chr2:g.29222407-29222407:G>A(GRCh38). Only ClinVar reports this variation, classified as Pathogenic, Risk Factor, Likely Benign, and Uncertain Significance for Neuroblastoma-related phenotypes. There are obvious contradictions in the role of this variation, making it difficult to determine whether the Neuroblastoma presented by both patients has a genetic origin or not. This case demonstrates how a lack of concordance, not only among data sources but also within the information provided by the same data source, complicates the correct diagnosis of patients. Patients Onco-3 and Onco-4 have osteosarcoma and the same mutation: Chr14:g.104780214-104780214:C>T(GRCh38). This variant is exclusively reported by ClinVar and is classified as likely pathogenic. Because none of the data sources are complete, clinicians should use a combination of them to make accurate genomic-based diagnoses.

Then we focused on the five patients who suffer from a cardiology Disorder. Cardio-1 and Cardio-2 patients are affected by arrhythmogenic cardiomyopathy. The Chr18:g.31542655-31542655:G>A variation, identified in Cardio-1 patient, is classified as Benign by ClinVar but as Pathogenic according to LOVD, indicating a major discrepancy. The Chr12:g.32896656-32896656:C>T variation, identified in Cardio-2 patient, is classified as Uncertain Significance by LOVD and Benign by ClinVar. These circumstances make it extremely difficult to assess whether a patient’s cardiac disorder has a genetic origin.

Cardio-3, Cardio-4, and Cardio-5 patients are affected by Hypertrophic Cardiomyopathy. The information associated with the Chr11:g.47360070-47360070:C>T variation, identified in Cardio-3 patient, is concordant because it is classified as Pathogenic by both ClinVar and CardioDB. Cardio-4 patient has a variation classified as Uncertain Significance in ClinVar and as Pathogenic in LOVD. These differences between the classifications provided by LOVD and ClinVar complicate the clinical diagnosis of this patient. The same happens to Cardio-5 patient. In that case, the concordance issue occurs between ClinVar and CardioDB. The Chr14:g.23417598-23417598:C>T variation was identified in this patient, classified as Likely Pathogenic in ClinVar and as Uncertain Significance in CardioDB.

## Discussion

Analyzing the completeness and concordance of information about DNA variations in six well-known data sources allowed us to characterize the big data problem that challenges genomics-based clinical diagnosis in greater detail.

Regarding the study of completeness at the schema level, there is a high degree of heterogeneity between data sources that use an "interpretation" approach (i.e., geneticists determine whether the variation causes or does not cause the disorder) and those that use a "statistical metrics" approach (i.e., the strength of the association between the variation and the disorder is calculated). When it comes to data sources that use the same approach, their structures and semantics differ significantly. As a result, data analyses like the one performed in this work are complex. These issues were addressed by creating a conceptual schema based on the CSHG, which guided the characterization of information provided by heterogeneous data sources and improved semantic interoperability and data integration processes.

In terms of the data completeness metric, there is a significant difference in the variations provided by each data source.

- ClinVar and Ensembl offer variations for every oncology and cardiology disorder. The reason ClinVar provides such a large number of variations is that it has been widely adopted by genomics professionals and that the most popular variant interpretation standards encourage using it [26]. However, Ensembl only provides that degree of coverage because nourishes from ClinVar.
- LOVD reports a few variations associated with oncology disorders. Furthermore, no variants for Osteosarcoma or Retinoblastoma have been reported. Even though LOVD is a collection of gene-based data sources with no limitations on the diseases that can be studied, oncology experts appear to prefer reporting their findings to other data sources rather than LOVD.
- GWAS Catalog only provides data on two cardiology disorders (Dilated and Hypertrophic Cardiomyopathy), even though it is a general-purpose data source that can report on several forms of cardiovascular disorders and many others. The disparity in prevalence rates of the disorders appears to be the cause of this. GWAS studies require a large enough sample of well-characterized patients to produce statistically significant evidence, meaning they are more feasible for more prevalent disorders. Dilated and Hypertrophic Cardiomyopathies are the most common, with prevalence rates of 1:500 and 1:2500, respectively. Still, other types of cardiomyopathies, such as Arrhythmogenic Cardiomyopathy, have an incidence of 1:1000 to 1:5000, and restrictive cardiomyopathy is the least common form of this condition in the population, representing 2 to 5% of all cases. Furthermore, some less common forms of cardiomyopathy, such as Arrhythmogenic Cardiomyopathy, tend to coexist with more common ones like Dilated Cardiomyopathy [27].
- Despite the fact that CIViC focuses on cancer-related variations, it does not report on variants associated with Neuroblastoma, Retinoblastoma, and Osteosarcoma oncology disorders. One possible reason is that these cancers has a very low incidence (7.3% for Neuroblastoma, 2.8% for Retinoblastoma, and 2.4% for Osteosarcoma [28]), and CIViC contributors concentrate their efforts on more common cancers. For instance, CIViC reports variants associated with Acute Myeloid Leukemia and Acute Lymphoblastic Leukemia, both which have a much higher incidence (i.e., 30% [28]).
- CardioDB does not report on variations associated with Restrictive Cardiomyopathy or Short QT Syndrome. This is because CardioDB data was obtained from a study comprising 7,855 cardiomyopathy cases, and none suffered from Restrictive Cardiomyopathy or Short QT Syndrome. However, its relevance for the remaining conditions (i.e., Hypertrophic Cardiomyopathy, Dilated Cardiomyopathy, and Arrhythmogenic Cardiomyopathy) is significant.

Table 10 summarizes the CDL results for oncology and cardiology disorders. ClinVar is the most comprehensive database for both types of disorders. Although the CDL of the other data sources is significantly lower, they still contain unique variations. For instance, there are 30 variations associated with the oncology disorder Acute Myeloid Leukemia (4 in CIViC, 10 in Ensembl, 14 in LOVD, and 2 in GWAS Catalog) not reported by ClinVar, and sixteen of them have been reported to be disorder-causing. This demonstrates the importance of not focusing solely on ClinVar, as necessary information for clinical diagnosis will be missed.

**Table 10:** Results for the completeness at data level dimension per group of disorder

Database	Number of variations	CDL (%)
Oncology disorders		
ClinVar	4,599	94.33%
Ensembl	3,047	62.50%
GWAS Catalog	225	4.62%
LOVD	38	0.78%
CIViC	18	0.37%
Cardiology disorders		
ClinVar	52,052	92.88%
Ensembl	13,250	23.64%
GWAS Catalog	167	0.30%
LOVD	1,442	2.57%
CardioDB	1,649	2.94%

The results of our concordance analysis show that the information available in the studied data sources is highly concordant. Our findings support previous research on the consistency of interpretations of variations reported in ClinVar. Harrison et al. examined 244 variations and discovered that 87.6% of them had a majority consensus [29]. Yang et al. also examined the ClinVar classifications of 27,224 variations [30]. They found that, in general, a majority consensus was reached on nearly 90% of variations. However, the percentage of cardiology-related variations with majority consensus fell to 85%. These findings are consistent with ours (92.88% concordance for cardiology disorders).

Despite the high percentage of concordance for variations, hundreds of variations with discordant interpretations are reported. A situation like this must be carefully considered. Indeed, 93% of clinical experts claim to have encountered a variation with ambiguous interpretation [31]. This requires experts to invest additional time and effort to analyze those variations, resulting in delays in therapeutic decisions and actions.

Table 11 summarizes the concordance results for oncology and cardiology disorders. Although only 0.94% of variations associated with cardiology disorders are discordant, this percentage still includes 510 variations. Compared to oncology disorders, cardiology disorders have more reported variations as well as more discordant interpretations. This suggests that as more variations are linked to a disease, their concordance decreases, which is a challenge in this rapidly expanding domain that has yet to be solved.

Concordance issues have a direct impact on clinical care because different experts evaluate these conflicts differently [32]. This is consistent with the findings reported in Use case, which revealed that even a tiny fraction of discordant information makes it difficult to diagnose patients.

**Table 11:** Results for the concordance dimension per group of disorder

Database	Number of variations	Concordance (%)
Oncology Disorder		
Concordant Interpretations	4,879	99.82%
Discordant Interpretations	9	0.18%
Concordant GWAS	226	99.56%
Discordant GWAS	1	0.44%
Cardiology disorders		
Concordant Interpretations	53,685	99.06%
Discordant Interpretations	510	0.94%
Concordant GWAS	68	100.00%
Discordant GWAS	0	0.00 %

## Conclusions

The completeness and concordance of six well-known genomic data sources (i.e., ClinVar, Ensembl, GWAS Catalog, LOVD, CardioDB, and CIViC) in the context of two groups of disorders (cardiology and oncology disorders) were examined in the present research. Our findings show that relying solely on the information provided by some of these data sources is insufficient, and that there are concordance issues among this information.

Complete and consistent data is required to provide an accurate genomics-based diagnosis. However, assessing the completeness and concordance of genomics data is a time-consuming and labor-intensive task that is ignored too frequently. This is due to the immense amount of available data, the constant evolution of genomic knowledge, the dispersion of genomics data across hundreds of data sources, and the lack of interoperability. As a result, future work will focus on systematizing the process described in this work to evaluate the completeness and concordance of genomics data associated with a given disorder. As a result of this systematization, more and more insights will be generated, taking into account additional data sources and groups of disorders. An additional line of research is to evaluate other DQ metrics. This study evaluated those DQ metrics that more accurately depict the effects of just taking into account a small number of data sources for interpreting genomics variations. However, to assess the utility of each data source in a specific context, it may be crucial to examine more DQ metrics (see Table 3).

## Data Availability

Data and materials are publicly available at https://doi.org/10.5281/zenodo.7461844.

## Abbreviations

CDL: Completeness at the Data Level
CI: Confidence Interval
CSL: Completeness at the Schema Level
CSHG: Conceptual Schema of the Human Genome
DQ: Data Quality
ETL: Extract, Transform, Load
GWAS: Genome Wide Association Study
HPO: Human Phenotype Ontology
ISGE: Identify, Select, GEnerate
NGS: Next Generation Sequencing
